# A multivariate process quality correlation diagnosis method based on grouping technique

**DOI:** 10.1038/s41598-024-61954-y

**Published:** 2024-06-08

**Authors:** Qing Niu, Shujie Cheng, Zeyang Qiu

**Affiliations:** Department of Product Design, Lanzhou Jiaotong University, Lanzhou, Gansu People’s Republic of China

**Keywords:** Multivariate process quality, Correlation diagnosis, Grouping technology, Factor analysis, T^2^ control chart, Mechanical engineering, Applied mathematics

## Abstract

Correlation diagnosis in multivariate process quality management is an important and challenging issue. In this paper, a new diagnostic method based on quality component grouping is proposed. Firstly, three theorems describing the properties of the covariance matrix of multivariate process quality are established based on the statistical viewpoint of product quality, to prove the correlation decomposition theorem, which decomposes the correlation of all the quality components into a series of correlations of components pairs, and then by using the factor analysis method, all quality components are grouped in order to maximize the correlations in the same groups and minimize the ones between different groups. Finally, on the basis of correlations between different groups are ignored, T^2^ control charts of component pairs in the same groups are established to form the diagnostic model. Theoretical analysis and practice prove that for the multivariate process quality whose the correlations between different components vary considerably, the grouping technique enables the size of the correlation diagnostic model to be drastically reduced, thus allowing the proposed method can be used as a generalized theoretical model for the correlation diagnosis.

## Introduction

With the development of the modern global market, the product's quality has been one of the key factors that greatly influence the competitiveness of enterprises. In the whole formation of the product's quality, process quality is one of the most basic sessions because the product's quality will be influenced by every process' quality directly or indirectly, so process quality control is the essence of quality management in manufacturing.

The objective of managing univariate process quality was achieved by using *Shewhart's* control chart, which is a tool in the theory of statistical process control (SPC)^1–4^. But in modern manufacturing, there are many processes that involve more than one quality component. Due to the correlation of quality components, all components and their correlation must be monitored simultaneously^5,6^. The theory of monitoring the correlation shift of all the quality components using T^2^ control charts was originally proposed by *Hotelling*^7^*.* For a *p*-dimensional process quality ***y*** = (*y*_1_, *y*_2_,…, *y*_*p*_)^*T*^, the T^2^ statistic is defined as:

1 $$T^{2} = ({\mathbf{y}} - {{\varvec{\upmu}}})^{T} {{\varvec{\Sigma}}}^{ - 1} ({\mathbf{y}} - {{\varvec{\upmu}}})$$

where ***μ*** is the mean vector, and ***Σ*** is the covariance matrix of ***y***. When T^2^ > 0, it signifies that all the quality components in ***y*** are correlated.

The general distribution of T^2^ statistics can have different forms^8–10^. Particularly, when ***y*** follows the normal distribution *N*(***μ***, ***Σ***), the T^2^ statistic follows the *χ*^*2*^ distribution with *p*-freedom, and the proof can be found in Supplementary. Suppose *α* is the false probability, then the upper control limit (UCL) of the T^2^ statistic is $\chi_{\alpha }^{2} (p)$, and the lower control limit (LCL) is 0. Thus, the T^2^ control chart can be established to monitor the correlation shift of ***y***. T^2^ control chart has the advantage of being able to fully take into account the correlation between components and gives accurate false probability under condition of component correlation, however, this control chart is unable to pinpoint the cause(s) of the correlation shift when it is out of control. Since then, on the basis of T^2^ statistic, scholars have carried out a lot of research on the diagnostic methods of abnormal correlation shift between quality components, and have successively proposed diagnostic methods based on component combinations, principal component analysis, orthogonal decomposition of the T^2^ statistics, and intelligent diagnostic methods.

### Diagnosis method based on component combinations

For a *p*-dimensional process quality ***y*** = (*y*_1_, *y*_2_,…, *y*_*p*_)^*T*^, when the T^2^ control chart *K*, which monitors the correlation shift of all the quality components shows an abnormality, it indicates that the correlations of one or more quality component combinations must be abnormal. In order to diagnose the specific component combinations that cause the T^2^ control chart *K* to be abnormal, a straightforward approach is to use the exhaustive method, i.e., to list all possible forms of component combinations, and for each form of combinations, to create a T^2^ control chart. When the T^2^ control chart *K* displays an abnormality, the specific component combinations that lead to the abnormality of the T^2^ control chart *K* can be determined by analyzing the results of the T^2^ control charts corresponding to all combinations of quality components one by one^11–13^, this approach is referred as component combinations based diagnostic (CCBD) method in this paper. While this approach is theoretically sound and appealing, it has inherent deficiencies. For a *p*-dimensional process quality ***y*** = (*y*_1_, *y*_2_, …, *y*_*p*_)^*T*^, the number of T^2^ control charts using this method is $N = C_{p}^{2} + C_{p}^{3} + \cdots + C_{p}^{p}$ = 2^*p*^-*p*-1, where *N* is an exponential function of *p*, and the space complexity is *O*(2^*p*^). When *p* is small, this approach has some feasibility, however, when *p* increases, *N* will increase sharply, leading to a significant expansion of the diagnostic system scale, so this method is difficult to apply in practice.

On the other hand, the defect of information redundancy in diagnostic results can not be avoided in the CCBD method. For example, in a 4-dimensional process quality ***y*** = (*y*_1_, *y*_2_, *y*_3_, *y*_4_)^*T*^, suppose the abnormal correlation shift between *y*_1_ and *y*_2_ is the only cause which causes the correlation of ***y*** out of control. Now in the CCBD method, besides T^2^ control chart to monitor the correlation shift of (*y*_1_, *y*_2_) is out of control, the other combinations which contain *y*_1_ and *y*_2_, namely (*y*_1_, *y*_2_, *y*_3_) and (*y*_1_, *y*_2_, *y*_4_), their T^2^ control charts are both out of control. This phenomenon that because of the correlation shift of one component combination is out of control, the correlations of other component combinations which contain the abnormal component combination are all out of control is called as the redundancy of diagnostic messages. The redundancy in diagnostic results is disturbance for process quality adjustment.

### Diagnosis method based on principal component analysis

When the number of quality components to be monitored in manufacturing process is large, direct analysis of process quality data will lead to a significant increase in the computational effort of the diagnostic process. Therefore, reducing the complexity of process quality data in an appropriate means is an effective way to improve diagnostic efficiency. Because the principal component analysis (PCA) is a useful tool for dealing with high-dimensional data, scholars proposed by using the principal component analysis method^14–17^, the original process quality ***y*** = (*y*_1_, *y*_2_,…, *y*_*p*_)^*T*^ is converted to *p* independent principal components and sorted by variance decreasing order, denoted as ***z*** = (*z*_1_, *z*_2_, … , *z*_*p*_)^*T*^. Then firstly, *p Shewhart* control charts are constructed to monitor the normality of *z*_*i*_; Secondly, the first *n*(*n* < *p*) principal components whose the cumulative sum of their variance exceeds a specified critical value are grouped as component pairs, and T^2^ control charts are constructed to monitor the normality of (*z*_*i*_, *z*_*j*_) (*i*, *j* ≤ *n*, *i* ≠ *j*); At last, the normality of the rest principal components group (*z*_*n*+1_, *z*_*n*+2_,…, *z*_*p*_) is monitored by a T^2^ control chart.

Compared with the CCBD method, the number of control charts based on PAC method is $N = p + C_{n}^{2} + 1 = p + n(n - 1)/2 + 1$, the space complexity approximately is *O*(*p*^2^), and the diagnostic efficiency is improved. However, *n* still increases rapidly while *p* is increasing, the scale of diagnostic system is still large. Furthermore, due to *z*_*i*_ generally has no engineering meaning after conversion, the cause(s) which cause the correlation shift of ***y*** out of control can only be specified by a comprehensive analysis of all the results in control charts and consulting the mapping relationship between ***y*** and ***z***, the calculation of diagnosis is increased, and the accuracy of diagnostic results is affected. Meanwhile, the redundancy of diagnostic messages also can not be avoided.

### Diagnosis method based on correlation orthogonal decomposition

In 1995, *Mason*, *Young* and *Tracy*^18–20^ proposed by using regression analysis method, the T^2^ statistic can be decomposed into conditional and unconditional terms which have equal weight in the decomposition results and are orthogonally independent each other. Then, according to the statistical distribution of the conditional and unconditional terms, the corresponding control limits are established to diagnose the specific cause(s) when the manufacturing process is abnormal. Compared with the diagnostic methods based on principal component analysis, the conditional and unconditional terms obtained by MYT orthogonal decomposition method can be directly corresponded to the quality components or component combinations, which improves the accuracy of the diagnostic results.

As an example, in bivariate process quality ***y*** = (*y*_1_, *y*_2_)^*T*^, the basic idea of the MYT orthogonal decomposition method^21–24^ is to decompose the T^2^ statistic into the following form:

2 $$T^{2} = T_{1}^{2} + T_{2 \cdot 1}^{2}$$

where $T_{1}^{2}$, called the unconditional term, is related only to the quality component *y*_1_ and is used to measure the contribution shift in *y*_1_ to the T^2^ statistic; and $T_{2 \bullet 1}^{2}$, called the conditional term, whose value is related to the conditional probability *P*(*y*_2_|*y*_1_) and is used to measure the contribution in the correlation between *y*_1_ and *y*_2_ to the T^2^ statistic.

Similar to Eq. (2), the T^2^ statistic can also be decomposed into another form:

3 $$T^{2} = T_{2}^{2} + T_{1 \cdot 2}^{2}$$

where the unconditional term $T_{2}^{2}$ is related only to the quality component *y*_2_, and is used to measure the contribution shift in *y*_2_ to the T^2^ statistic; the conditional term $T_{1 \bullet 2}^{2}$ depends on the conditional probability* P*(*y*_1_|*y*_2_), and is used to measure the contribution in the correlation between *y*_2_ and *y*_1_ to the T^2^ statistic.

Conditional probability *P*(*y*_2_|*y*_1_) ≠ *P*(*y*_1_|*y*_2_) when *y*_1_ and *y*_2_ are correlated, and hence the conditional term $T_{2 \cdot 1}^{2} \ne T_{1 \cdot 2}^{2}$. For this reason, Eqs. (2) and (3) are two distinct representations of the T^2^ statistic's decomposition results. In general, for a *p*-dimensional process quality*** y*** = (*y*_1_, *y*_2_,…, *y*_*p*_)^*T*^, the decomposition results have a total of *p*(*p-*1)*…* × 2 × 1, and the space complexity is *O*(*p*!). As the number of quality components increases, under the condition that every possible form of decomposition is analyzed, will lead to a significant increase of calculations and a serious reduction in diagnostic efficiency. At the same time, the accuracy of the diagnostic results based on this method will be affected when there are obvious correlations between different quality components.

### Intelligent diagnosis methods

In addition to the traditional diagnostic methods based on mathematical model analysis, in recent years, with the development of artificial intelligence technology, intelligent diagnostic methods are applied to the field of multivariate process quality diagnosis, and the diagnostic methods based on artificial neural network (ANN)^25–28^, Bayesian network^29–32^, support vector machine (SVM)^33–35^, etc. have been widely applied. Intelligent diagnostic methods can effectively reduce the scale of the diagnostic system and improve the diagnostic efficiency, however, these methods generally require a large amount of data to train the network's parameters, and the constructed network are generally suitable for specific applications, thus their generality will be greatly restricted. Therefore, establish a general and efficient method for multivariate process quality correlation diagnosis is a major problem to be solved in the field of quality management.

### Sketch of the algorithm

In this paper, a new correlation diagnosis method based on quality component grouping is proposed. For the multivariate process quality ***y*** = (*y*_1_, *y*_2_,…, *y*_*p*_)^*T*^, three theorems describing the properties of the multivariate process quality covariance matrix are first established based on the statistical viewpoint of product quality in manufacturing processes; Then the correlation decomposition theorem is proved by drawing on the idea of decomposing the T^2^ statistic in the MYT orthogonal decomposition method, which decomposes the correlation of all the quality components into the correlations of all the component pairs, to reduce the space complexity of the diagnostic system to *O*(*p*^2^); Next, refer to the grouping idea in the principal component analysis method, based on the correlation between different components, the quality components are grouped, so that the correlations between components in the same groups are as large as possible, and the correlations between components of different groups are as small as possible; Finally, draw on the principle of component combination diagnosis method, on the premise of ignoring the correlations between different groups, quality components in the same groups are combined as component pairs to establish the corresponding T^2^ control charts, which constitutes the multivariate process quality correlation diagnostic system, thus the space complexity of the diagnostic system is reduced to approximate *O*(*p*), to improve the diagnostic efficiency.

## Covariance matrix properties of multivariate process quality

In the manufacturing process, factors affecting the product's quality can be attributed to 5 aspects: man, machines, materials, methods and environment (4M1E). On this basis, ISO9000 supplemented another 3 factors: the manufacturing software, auxiliary materials and utilities. Among the many factors affecting the product's quality, changes in any one of them will have an impact on the final quality of the product, so the product's quality is fluctuating in manufacturing. Tolerance theory is a direct proof of the fluctuation of the product's quality.

For the multivariate process quality ***y*** = (*y*_1_, *y*_2_,…, *y*_*p*_)^*T*^, the covariance matrix is an important parameter to describe its correlation. Combined with the fluctuation of the product's quality in the manufacturing process, this paper firstly establishes 3 theorems describing the characteristics of the covariance matrix of multivariate process quality.

### Theorem 1

In the covariance matrix ***Σ*** of the multivariate process quality ***y*** = (*y*_1_, *y*_2_,…, *y*_*p*_)^*T*^, all of the elements are not 0.

Suppose the mean vector of ***y*** is ***μ* = **(*μ*_1_, *μ*_2_,…, *μ*_*p*_)^*T*^. According to the definition of the covariance matrix, it is known that:

4 $$\begin{aligned} {{\varvec{\Sigma}}} & = \left[ {\begin{array}{*{20}c} {E[(y_{1} - \mu_{1} )(y_{1} - \mu_{1} )]} & {E[(y_{1} - \mu_{1} )(y_{2} - \mu_{2} )]} & \cdots & {E[(y_{1} - \mu_{1} )(y_{p} - \mu_{p} )]} \\ {E[(y_{2} - \mu_{2} )(y_{1} - \mu_{1} )]} & {E[(y_{2} - \mu_{2} )(y_{2} - \mu_{2} )]} & \cdots & {E[(y_{2} - \mu_{2} )(y_{p} - \mu_{p} )]} \\ \vdots & \vdots & \vdots & \vdots \\ {E[(y_{p} - \mu_{p} )(y_{1} - \mu_{1} )]} & {E[(y_{p} - \mu_{p} )(y_{2} - \mu_{2} )]} & \cdots & {E[(y_{p} - \mu_{p} )(y_{p} - \mu_{p} )]} \\ \end{array} } \right] \\ & = \left[ {\begin{array}{*{20}l} {\sigma_{11} } \hfill & {\sigma_{12} } \hfill & \cdots \hfill & {\sigma_{1p} } \hfill \\ {\sigma_{21} } \hfill & {\sigma_{21} } \hfill & \ldots \hfill & {\sigma_{2p} } \hfill \\ \vdots \hfill & \vdots \hfill & \vdots \hfill & \vdots \hfill \\ {\sigma_{p1} } \hfill & {\sigma_{p1} } \hfill & \cdots \hfill & {\sigma_{pp} } \hfill \\ \end{array} } \right] \\ \end{aligned}$$

For any element $\sigma_{ij} = E[(y_{i} - \mu_{i} )(y_{j} - \mu_{j} )]$ in ***Σ***, the sufficient and necessary condition for it to be 0 is:

5 $$y_{i} = \mu_{i} \quad {\text{or}} \quad y_{j} = \mu_{j}$$

According to the properties of mathematical expectation, Eq. (5) implies that the quality component *y*_*i*_ or *y*_*j*_ is a constant in the manufacturing process. Clearly, this is in conflict with the viewpoint of the fluctuation of the product's quality, and therefore, Eq. (5) does not hold, i.e., all the elements in ***Σ*** are not 0.

### Theorem 2

The covariance matrix ***Σ*** of the multivariate process quality ***y*** = (*y*_1_, *y*_2_, … , *y*_*p*_)^*T*^ is a real symmetric positive definite matrix.

According to Eq. (4) on the definition of the covariance matrix:

$$\begin{aligned} & \sigma_{ij} = E[(y_{i} - \mu_{i} )(y_{j} - \mu_{j} )] \\ & \sigma_{ji} = E[(y_{j} - \mu_{j} )(y_{i} - \mu_{i} )] \\ \end{aligned}$$

From the properties of mathematical expectation can be seen:

$$\sigma_{ij} = \sigma_{ji}$$

That is, ***Σ*** is a symmetric matrix.

Let *p*-dimensional vector ***c*** = (*c*_1_, *c*_2_,…, *c*_*p*_)^*T*^ ≠ **0**.

6 $${\mathbf{c}}^{T} {\mathbf{\Sigma c}} = (c_{1} ,c_{2} , \cdots c_{p} ){{\varvec{\Sigma}}}(c_{1} ,c_{2} , \cdots c_{p} )^{T}$$

Bringing Eq. (4) into (6), after simplification and consolidation, we get

7 $${\mathbf{c}}^{T} {\mathbf{\Sigma c}} = E\left[ {\left( {\sum\limits_{i = 1}^{p} {c_{i} (y_{i} - \mu_{i} )} } \right)\left( {\sum\limits_{k = 1}^{p} {(y_{k} - \mu_{k} )c_{k} } } \right)} \right]$$

Let random variable $z = \sum\nolimits_{i = 1}^{p} {c_{i} (y_{i} - \mu_{i} )}$, bringing this into Eq. (7), we get

$${\mathbf{c}}^{T} {\mathbf{\Sigma c}} = E(z^{2} ) \ge 0$$

From the proof of Theorem 1, it is clear that according to the viewpoint of the fluctuation of the product's quality, *z* ≠ 0, i.e.

$${\mathbf{c}}^{T} {\mathbf{\Sigma c}} = E(z^{2} ) > 0$$

Therefore, the covariance matrix ***Σ*** of the multivariate process quality ***y*** = (*y*_1_, *y*_2_,…, *y*_*p*_)^*T*^ is a real symmetric positive definite matrix.

### Theorem 3

The inverse matrix ***Σ***^−1^ of the covariance matrix ***Σ*** of the multivariate process quality ***y*** = (*y*_1_, *y*_2_,…, *y*_*p*_)^*T*^ is a real symmetric positive definite matrix.

First prove the symmetry of ***Σ***^−1^. It follows from the symmetry of ***Σ***:

$${{\varvec{\Sigma}}} = {{\varvec{\Sigma}}}^{T}$$

Inverting both ends of the above equation:

$${{\varvec{\Sigma}}}_{{}}^{ - 1} = ({{\varvec{\Sigma}}}_{{}}^{T} )^{ - 1} = ({{\varvec{\Sigma}}}_{{}}^{ - 1} )^{T}$$

The above equation shows that ***Σ***^−1^ is a symmetric matrix.

Let the eigenvalues of ***Σ*** be *λ*_1_, *λ*_2_,…, *λ*_*p*_. By the positive definiteness of ***Σ***, *λ*_*i*_ > 0 (*i* ≤ *p*). According to the nature of the inverse matrix, the eigenvalues of ***Σ***^−1^ are 1/*λ*_1_, 1/*λ*_2_,…, 1/*λ*_*p*_, i.e., the eigenvalues of ***Σ***^−1^ are all greater than 0, so ***Σ***^−1^ is a positive definite matrix.

## Theoretical basis for correlation grouping diagnosis

The exponential function between *N* and *p* is the main reason why applying this approach is difficult in the CCBD method. If the gradient of *N* with *p* can be lowered by proper means, the defect of diagnostic system scale expands greatly while *p* is increasing will be avoided to a certain extent, and thus this approach can be applied in multivariate process quality management.

### Correlation decomposition

#### Theorem 4

In the multivariate process quality ***y*** = (*y*_1_, *y*_2_,…, *y*_*p*_)^*T*^, the sufficient and necessary condition of the correlation of all the components exists is, for any two components *y*_*i*_ and *y*_*j*_, they are correlated.

Firstly, the sufficiency of Theorem 4 is proved. Any two components *y*_*i*_ and *y*_*j*_ in ***y*** are correlated shows that *σ*_*ij*_ ≠ 0. From Theorems 2 and 3, the covariance matrix ***Σ*** and its inverse matrix ***Σ***^−1^ are real symmetric positive definite matrix. From the definition of the T^2^ statistic in Eq. (1), it is clear that for any sample data, its T^2^ statistic is greater than 0, i.e., the correlation of all the components exists.

The following proves the necessity of Theorem 4 by reduction and absurdum. The existence of correlation of all the components in ***y*** implies that for any sample data, its T^2^ statistic is greater than 0. From the definition of the T^2^ statistic in Eq. (1), there exists an inverse matrix of the covariance matrix ***Σ*** of ***y***, and the rank of ***Σ*** is *p.*

8 $$R({{\varvec{\Sigma}}}) = p$$

Assume *y*_*k*_ and *y*_*j*_ in ***y*** are uncorrelated, i.e., *σ*_*kj*_ = 0. By the definition of covariance, there is:

9 $$\sigma_{kj} = E[(y_{k} - \mu_{k} )(y_{j} - \mu_{j} )] = 0$$

The sufficient and necessary condition for Eq. (9) to hold is *y*_*k*_ = *μ*_*k*_ or *y*_*j*_ = *μ*_*j*_. It may be useful to set *y*_*k*_ = *µ*_*k*_. From the definition of covariance, we know that for any component *y*_*i*_ (*i* = 1, 2 ,… , *p*), there are:

10 $$\sigma_{ki} = E[(y_{k} - \mu_{k} )(y_{i} - \mu_{i} )] = 0$$

Equation (10) shows that in the covariance matrix ***Σ*** of ***y***, the *k*th row and *k*th column are both 0, i.e., *R*(***Σ***) ≤ *p *− 1. This contradicts Eq. (8), the assumption is not valid, and the necessity of Theorem 4 is proved.

Theorem 4 means that the correlation of all the quality components can be represented as correlations of component pairs, so in the correlation diagnostic system, it only needs to monitor the correlation shifts of all the component pairs. In addition, T^2^ control chart to monitor the correlation shift of all the components should be added, the number of T^2^ control charts is *N* = *C*_*p*_^*2*^ + 1 = *p(p *− 1)/2 + 1, *N* is the power function of *p*, the space complexity of the diagnostic system is lowered to *O*(*p*^*2*^). Compared to the CCBD method, the gradient of *N* with *p* is decreased significantly. Meanwhile, because the component pair is the minimum combination of components, the information redundancy in diagnostic results can be avoided effectively.

### Grouping principle

Although the functional relation between *N* and *p* is lowered to a power function by correlation decomposition, *N* will still increase rapidly while *p* is increasing, so further proper ways should be adopted to reduce the scale of the diagnostic system on the basis of the above analysis. For this reason, this paper proposes the following grouping principle.

#### Theorem 5

Let *p* = *p*_1_ + *p*_2_ + … + *p*_*m*_, where *p* and *p*_*i*_ (*i* = 1, 2, … , *m*) are integers greater than 0, *m* > 1. In this case there is the following inequality:

11 $$C_{p}^{2} > \sum\limits_{i = 1}^{m} {C_{{p_{i} }}^{2} }$$

The proof of Theorem 5 proceeds as follows:

$$\begin{aligned} & \sum\limits_{i = 1}^{m} {C_{{p_{i} }}^{2} = \sum\limits_{i = 1}^{m} {\frac{{p_{i} (p_{i} - 1)}}{2}} } \\ & \quad = \frac{1}{2}(\sum\limits_{i = 1}^{m} {p_{i}^{2} } - p) \\ & \quad < \frac{1}{2}(\sum\limits_{i = 1}^{m} {p_{i}^{2} } + 2\sum\limits_{\begin{subarray}{l} k,j = 1 \\ k \ne j \end{subarray} }^{m} {p_{k} p_{j} } - p) \\ & \quad = \frac{1}{2}((\sum\limits_{i = 1}^{m} {p_{i} } )^{2} - p) \\ & \quad = \frac{1}{2}(p^{2} - p) \\ & \quad = C_{p}^{2} \\ \end{aligned}$$

Theorem 5 shows that for the multivariate process quality ***y*** = (*y*_1_, *y*_2_,…, *y*_*p*_)^*T*^, if the quality components are grouped according to the degree of correlations, so that the correlations of quality components located within the same groups should be as large as possible, and the correlations of quality components located between different groups should be as small as possible, the number of T^2^ control charts in the diagnostic model can be further reduced by ignoring the correlations of the quality components located in the different groups, and the reduction of the number of T^2^ control charts is $$\sum\nolimits_{\begin{subarray}{l} k,j = 1 \\ k \ne j \end{subarray} }^{m} {p_{k} p_{j} }$$, where *m* is the number of quality components grouped, *p*_*k*_ and *p*_*j*_ denote the number of quality components contained in the *k*th and *j*th groups after grouping. In this case, the space complexity of the multivariate process quality correlation diagnostic model based on the grouping technique is approximated as *O*(*p*).

## Methodology for grouping quality components

Grouping techniques can lead to a significant reduction in the number of T^2^ control charts required in the correlation diagnostic system. Typically, quality components can be grouped with reference to practical experience, but this way can not give an accurate estimate of the error before and after grouping. In order to analyze the errors quantitatively, a grouping method based on the analysis of the covariance matrix of the quality components is used here.

Before grouping, the multivariate process quality ***y*** = (*y*_1_, *y*_2_,…, *y*_*p*_)^*T*^ needs to be standardized in order to avoid differences in the observed scales from affecting the grouping results:

12 $$y_{i}^{*} = \frac{{y_{i} - \mu_{i} }}{{\sigma_{i} }}$$

where *μ*_*i*_ and *σ*_*i*_ are the mean and variance of *y*_*i*_. In the standardization result ${\mathbf{y}}^{*} = (y_{1}^{*} ,y_{2}^{*} , \cdots ,y_{p}^{*} )^{T}$, the mean of each component is 0, and the variance is 1.

### Factor analysis

Factor analysis is a method of grouping components based on the degree of correlations between different components, using the covariance matrix of a random vector as a reference. The basic model of factor analysis is as follows^36,37^:

1. The standardized multivariate process quality y^*^ is an observable random vector with mean vector *E*(y^*^) = **0** and covariance matrix *D*(y^*^) = Σ^*^;
2. The common factor vector ***F*** = (*F*_1_, *F*_2_, …, *F*_*m*_)^*T*^ (*m* < *p*) is an unobservable random vector with mean vector *E*(***F***) = **0** and covariance matrix *D*(***F***) = ***I***, where ***I*** is a diagonal matrix where the main diagonal elements are 1, and the remaining elements are 0, i.e., the components in ***F*** are independent of each other;
3. The error vector ***ε*** = (*ε*_1_, *ε*_2_, …, *ε*_*p*_)^*T*^ is independent of the common factor vector ***F*** with *E*(***ε***) = **0**, and the covariance matrix ***D***(***ε***) is a diagonal matrix:
  $$D({{\varvec{\upvarepsilon}}}) = \left( {\begin{array}{*{20}c} {\sigma_{{\varepsilon_{1} }}^{2} } & {} & {} & {} \\ {} & {\sigma_{{\varepsilon_{2} }}^{2} } & {} & {} \\ {} & {} & \ddots & {} \\ {} & {} & {} & {\sigma_{{\varepsilon_{p} }}^{2} } \\ \end{array} } \right)$$

Under the above conditions, the factor analysis model can be expressed as the following equations:

13 $$\left\{ {\begin{array}{*{20}l} {y_{1}^{*} = a_{11} F_{1} + a_{12} F_{2} + \cdots + a_{1m} F_{m} + \varepsilon_{1} } \hfill \\ {y_{2}^{*} = a_{21} F_{1} + a_{22} F_{2} + \cdots + a_{2m} F_{m} + \varepsilon_{2} } \hfill \\ {\begin{array}{*{20}c} \vdots & {} & {} & {} \\ \end{array} } \hfill \\ {y_{p}^{*} = a_{p1} F_{1} + a_{p2} F_{2} + \cdots + a_{pm} F_{m} + \varepsilon_{m} } \hfill \\ \end{array} } \right.$$

Expressing the above system of equations in matrix form:

14 $${\mathbf{y}}^{*} = {\mathbf{AF}} + {{\varvec{\upvarepsilon}}}$$

where *a*_*ij*_ in the matrix ***A*** = (*a*_*ij*_)_*p*×*p*_ is called the factor loading, and its absolute value indicates the degree of dependence between the quality component $y_{i}^{*}$ and the common factor *F*_*j*_. The matrix ***A*** formed by all the factor loadings is called the factor loading matrix.

From Eq. (14), calculate the covariance matrix of y^*^:

15 $${{\varvec{\Sigma}}}^{*} = D({\mathbf{y}}^{*} ) = D({\mathbf{AF}}) + D({{\varvec{\upvarepsilon}}}) = {\mathbf{A}}D({\mathbf{F}}){\mathbf{A}}^{T} + D({{\varvec{\upvarepsilon}}}) = {\mathbf{AA}}^{T} + D({{\varvec{\upvarepsilon}}})$$

On the other hand, by Theorem 2, Σ^*^ is a real symmetric positive definite matrix for which *Cholesky* decomposition is performed:

16 $${{\varvec{\Sigma}}}^{*} = {\mathbf{GG}}^{T}$$

where ${\mathbf{G}} = (\sqrt {\lambda_{1} } {\mathbf{e}}_{1} ,\sqrt {\lambda_{2} } {\mathbf{e}}_{2} , \cdots ,\sqrt {\lambda_{p} } {\mathbf{e}}_{p} )$, *λ*_*i*_(*i* = 1, 2, …, *p*) are the eigenvalues of the covariance matrix Σ^*^ with *λ*_1_ > *λ*_2_ > … > *λ*_*p*_, ***e***_*i*_ is the eigenvector corresponding to *λ*_*i*_.

Comparing Eqs. (15) and (16), it can be seen that if ***A*** = ***G***, the error vector ***ε*** = **0** in Eq. (14), the obtained factor analysis model is accurate, but this means that after standardization, all the quality components in y^*^ will be grouped into *p* groups, i.e., the accurate factor analysis model can only be obtained when the correlations between the quality components in y^*^ are completely ignored. Therefore, considering the general situation, it is necessary to retain most of the correlations between the quality components, in which case an approximation of the factor loading matrix ***A*** is constructed from the first *m* (*m* < *p*) columns of the matrix ***G***, i.e.:

17 $${\mathbf{A}} \approx (\sqrt {\lambda_{1} } {\mathbf{e}}_{1} ,\sqrt {\lambda_{2} } {\mathbf{e}}_{2} , \cdots ,\sqrt {\lambda_{m} } {\mathbf{e}}_{m} )$$

### Error analysis

The error vector ***ε*** ≠ **0** when building the factor analysis model from the factor loading matrix derived from Eq. (17), this implies that there must be a certain amount of information loss when grouping the quality components in y^*^ based on Eq. (14).

In statistics, the total amount of information contained in a random variable is generally measured by its variance. In Eq. (14), let ***A*** = ***G***, which gives the sum of the variances of the components in y^*^ under the exact decomposition condition:

18 $$\sum\limits_{i = 1}^{p} {D(y_{i}^{*} } ) = \sum\limits_{i = 1}^{p} {\lambda_{i} }$$

Equation (18) shows that under the condition of exact decomposition, the sum of the information contained in all the quality components in y^*^ is equal to the cumulative sum of all the eigenvalues of the covariance matrix Σ^*^ of y^*^.

The factor loading matrix ***A*** is then constructed according to Eq. (17), at which point it is given by Eq. (14):

19 $$\sum\limits_{{{{i}} = {1}}}^{{{p}}} {{{D(y}}_{{{i}}}^{*} {)}} = \sum\limits_{{{{i}} = {1}}}^{{{m}}} {{\uplambda }_{{{i}}} } + \sum\limits_{{{{i}} = {1}}}^{{{p}}} {{{D(\varepsilon }}_{{{i}}} {)}}$$

Comparing Eq. (18) with Eq. (19) shows that grouping the quality components in y^*^ with Eq. (14), under the condition of ignoring the correlation of the quality components between different groups, the sum of information loss is $\sum\nolimits_{i = m + 1}^{p} {\lambda_{i} }$. Therefore, for a specified error *β*, the number of quality component group *m* can be determined by the following inequality:

20 $$\eta = \frac{{\sum\nolimits_{i = 1}^{m} {\lambda_{i} } }}{{\sum\nolimits_{i = 1}^{p} {\lambda_{i} } }} \ge 1 - \beta$$

where *η* is the cumulative variance contribution rate of the first *m* eigenvalues. Empirically, when *η* > 80% ~ 85%, the number of groupings *m* can be determined by inequality (20). The value of *η* can be reasonably adjusted in combination with specific applications, but the basic principle of adjustment is that it should be conducive to the reasonable interpretation of the factor analysis model.

### Correlation diagnostic algorithm based on grouping theory

The above analysis is founded on the condition that the mean *μ*_*j*_ and covariance matrix ***Σ*** of the manufacturing process are given. However, in many applications, these parameters are generally unknown. In this case, the unbiased estimator of the manufacturing process parameters can be calculated from a set of sample data ***y***_*i*_ = (*y*_*i*1_, *y*_*i*2_, … , *y*_*ip*_)^*T*^, (*i* = 1, 2,…, *n*) collected while the process is in stable state.

21 $$\mu_{j} = \frac{1}{n}\sum\limits_{i = 1}^{n} {y_{ij} } \quad \left( {j = 1,2, \ldots ,p} \right)$$

22 $$\sigma_{j} = \frac{1}{n - 1}\sum\limits_{i = 1}^{n} {(y_{ij} - \mu_{j} )^{2} } \quad \left( {j = 1,2, \ldots ,p} \right)$$

Then the sample data can be standardized as ${\mathbf{y}}_{i}^{*} = (y_{i1}^{*} ,y_{i2}^{*} , \cdots ,y_{ip}^{*} )^{T}$, (*i* = 1, 2,…, *n*), where

23 $$y_{ij}^{*} = \frac{{y_{ij} - \mu_{j} }}{{\sigma_{j} }}\quad \left( {j = 1,2, \ldots ,p} \right)$$

The covariance matrix Σ^*^ can be calculated by the standardize sample data:

24 $${{\varvec{\Sigma}}}^{*} = \frac{1}{n - 1}\sum\limits_{i = 1}^{n} {{\mathbf{y}}_{i}^{*} {\mathbf{y}}_{i}^{*T} }$$

Based on the above analysis, after grouping the quality components in the standardized multivariate process quality y^*^ using factor analysis method, on the premise of ignoring the correlation of quality components between different groups, the quality components within the same groups are combined as component pairs, and the corresponding binary T^2^ control charts are established to form the multivariate process quality correlation diagnostic model. The space complexity of this diagnostic model is approximated as a linear function of the quality component number *p*, which can lead to a significant improvement in the efficiency of the diagnosis.

The multivariate process quality correlation diagnostic model based on grouping technique can be constructed as follows:

1. Collect sufficient quality data ***y***_*i*_(*i* = 1, 2,…, *n*) while the manufacturing process is in stable state;
2. Calculate the manufacturing parameters according to Eqs. (21)–(24);
3. Calculate the eigenvalues of the covariance matrix Σ^*^ and arrange all the eigenvalues in descending order as $\lambda_{{1}} \, > \,\lambda_{{2}} \, > \, \cdots \, > \,\lambda_{p}$ ;
4. Calculate the eigenvector ***e***_*i*_ corresponding to the eigenvalue *λ*_*i*_(*i* = 1, 2, …, *p*);
5. For the given error *β*, calculate the number *m* of eigenvectors for constructing the factor loading matrix according to inequality (20), and then construct the factor loading matrix ***A*** from the first *m* eigenvectors according to Eq. (17);
6. Group all the quality components according to Eqs. (13), and the grouping results are recorded as *G*_1_, *G*_2_, …, *G*_*m*_;
7. For each pair of components $(y_{s}^{*} ,y_{t}^{*} )$ (*s* ≠ *t*) in *G*_*k*_ (*k* = 1, 2, …, *m*), build the corresponding T^2^ control chart *K*_*st*_;
8. Establish the T^2^ control chart *K* to monitor the correlation shift of all the quality components.

In the manufacturing process, if the T^2^ statistic of the new sample data exceeds the control limit in the control chart *K*, it indicates that the correlation shift of all the quality components is abnormal, and the cause(s) can be specified by examining the rest binary T^2^ control charts in the diagnostic model.

## Case study

**Figure 1:**
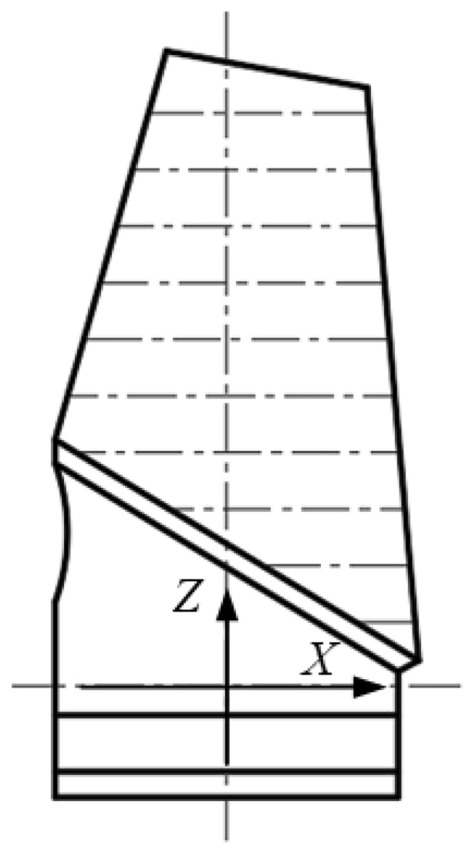
Contour method of blade inspection.

**Figure 2:**
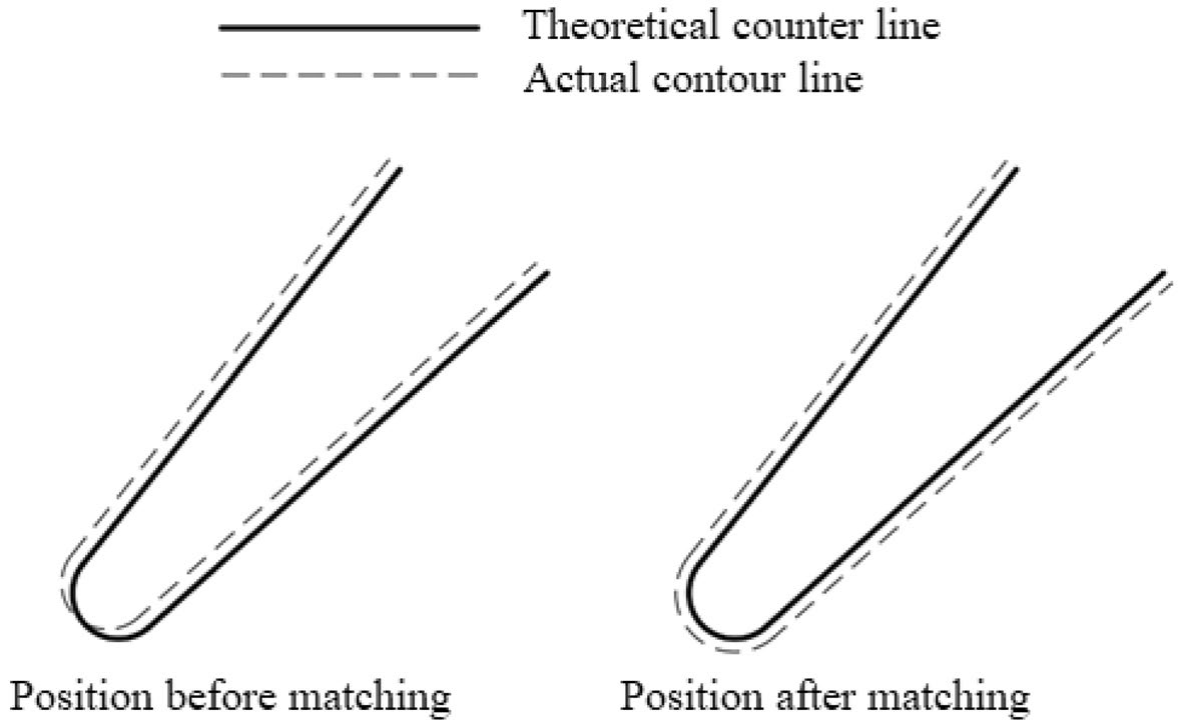
Theoretical and actual contour lines of the blade.

Blades are important parts in steam turbines and aviation engines, and their machining quality directly affects the life and performance of the equipment. The contour method is a commonly used blade quality inspection technique, and its basic principle is to measure a number of cross-section contour lines of the blade along the height direction (*Z*-axis direction) in the way shown in Fig. 1, and then match the actual contour lines measured in different height directions with their respective theoretical contour lines by translational and rotational transformations as shown in Fig. 2, so as to decompose the blade profiling error into 4 quality components: blade contouring error before matching, blade contouring error after matching, blade positional error, and blade torsion error.

The machining process shows that the 4 quality components are correlated, so it is necessary to monitor the correlation shift during the manufacturing process and to diagnose the causes of the abnormal correlation. Here, a T^2^ control chart is used to monitor the correlation shift of the 4 quality components, and the method proposed in this paper is used to diagnose the causes of the abnormal correlation.

### Parameters estimation

The above 4 quality components are expressed in vector form as ***y*** = (*y*_1_, *y*_2_, *y*_3_, *y*_4_)^*T*^. 15 sample data are collected at the cross-section height *Z* = 25 mm as shown in Table 1, in order to estimate the mean vector and covariance matrix for the manufacturing process.

**Table 1:** Sample data used for process parameter estimation.

No	Blade contouring error before matching (*y*_1_/mm)	Blade contouring error after matching (*y*_2_/mm)	Blade positional error (*y*_3_/mm)	Blade torsion error (*y*_4_/’)
1	0.083	0.043	0.098	2.018
2	0.086	0.045	0.099	2.216
3	0.084	0.045	0.098	2.239
4	0.077	0.036	0.093	2.108
5	0.076	0.034	0.091	2.156
6	0.081	0.042	0.095	2.332
7	0.078	0.037	0.093	2.259
8	0.087	0.046	0.101	2.184
9	0.085	0.044	0.099	2.247
10	0.089	0.051	0.105	2.318
11	0.075	0.034	0.089	2.287
12	0.088	0.047	0.104	2.047
13	0.074	0.033	0.086	2.275
14	0.082	0.041	0.098	2.066
15	0.080	0.041	0.093	2.307

Experience shows that the 4 quality components to be monitored generally follow normal distribution. In order to check the normality of the sample data, set the confidence level *α*_*t*_ = 0.95, and the *Shapiro*–*Wilk* test is done on the data of the 4 quality components in Table 1, and the results are shown in Table 2. It can be seen that the *W* statistics of the four components are all greater than the critical value *W*(15,0.05) = 0.881, indicating that the sample data in Table 1 follow normal distribution.

**Table 2:** Results of normality test for sample data.

Quality components	*W* statistics	Critical value *W*(15,0.05)
*y*_1_	0.9551	0.881
*y*_2_	0.9476	
*y*_3_	0.9707	
*y*_4_	0.9182	

**Figure 3:**
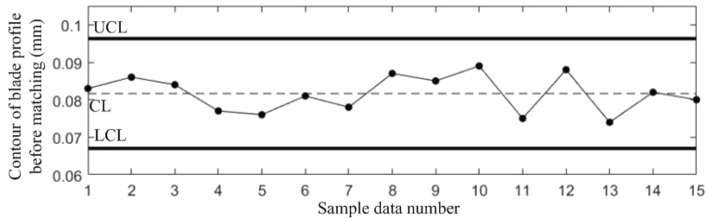
Shewhart control chart for sample data component *y*_1_.

**Figure 4:**
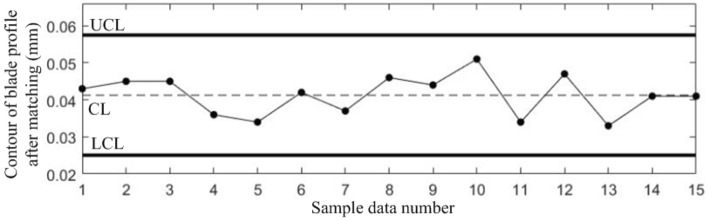
Shewhart control chart for sample data component *y*_2_.

**Figure 5:**
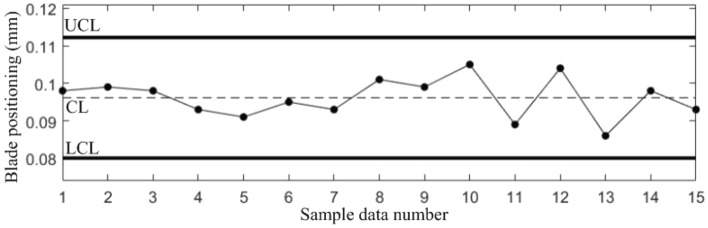
Shewhart control chart for sample data component *y*_3_.

**Figure 6:**
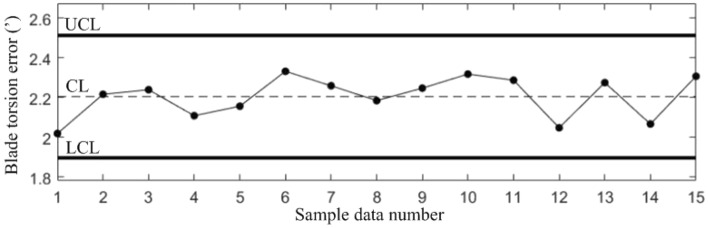
Shewhart control chart for sample data component *y*_4_.

**Figure 7:**
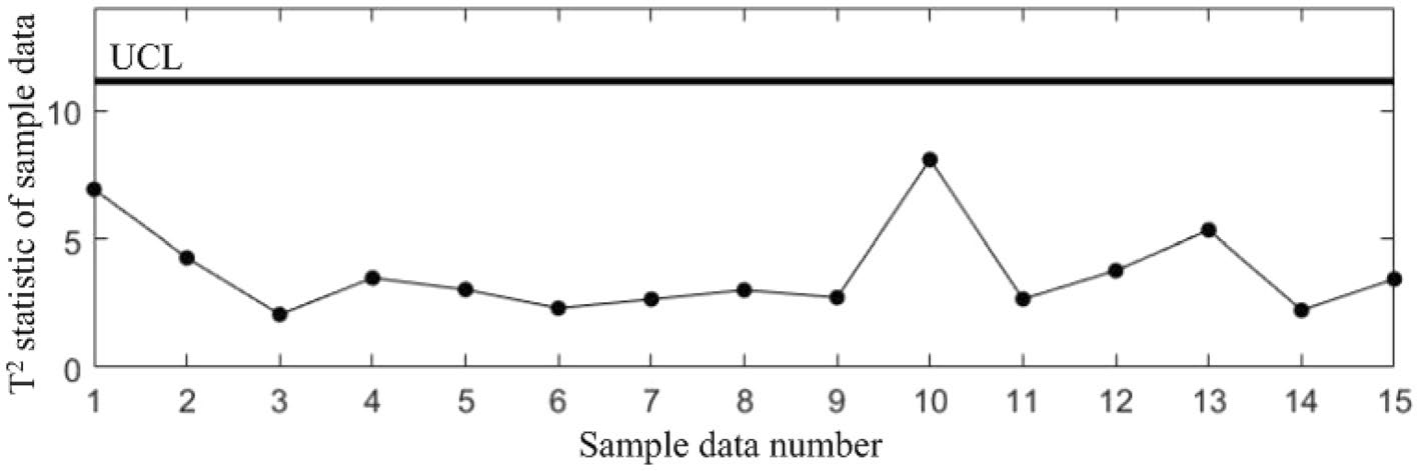
T^2^ control chart for sample data.

The sample data used to estimate the process parameters must be collected while the manufacturing process is in stable state, therefore, for the sample data in Table 1, the probability of false alarm *α* = 0.0027 is set for each quality component with reference to the 3*σ* principle of the *Shewhart* control chart. According to *Bonferroni* inequality and *χ*^2^ distribution, take the false alarm probability *α*_*y*_ = 0.025 of the correlation shift, and establish *Shewhart* control charts for the 4 quality components and T^2^ control chart to monitor the correlation shift, as shown in Figs. 3, 4, 5, 6 and 7.

Figures 3, 4, 5, 6 and 7 show that all the 5 control charts are in normal level, indicating that the sample data in Table 1 are obtained while the blade manufacturing process is in stable state, and can be used for process parameter estimation. The calculated mean vector and standard deviation are:

$$\begin{aligned} & {{\varvec{\upmu}}} = (0.0817,0.0413,0.0961,2.2039)^{T} \\ & {{\varvec{\upsigma}}} = (0.0029,0.0054,0.0054,0.1029)^{T} \\ \end{aligned}$$

The sample data in Table 1 are standardized and the results are shown in Table 3.

**Table 3:** Sample data after standardization.

No	y*_1_	y*_2_	y*_3_	y*_4_
1	0.2733	0.3207	0.3485	− 1.8061
2	0.8881	0.6908	0.5351	0.1172
3	0.4782	0.6908	0.3485	0.3406
4	− 0.9564	− 0.9745	− 0.5849	− 0.9319
5	− 1.1613	− 1.3445	− 0.9583	− 0.4656
6	− 0.1366	0.1357	− 0.2116	1.2440
7	− 0.7514	− 0.7895	− 0.5849	0.5349
8	1.0930	0.8758	0.9085	− 0.1936
9	0.6831	0.5057	0.5351	0.4183
10	1.5029	1.8009	1.6552	1.1080
11	− 1.3633	− 1.3445	− 1.3316	0.8069
12	1.2979	1.0608	1.4685	− 1.5244
13	− 1.5712	− 1.5296	− 1.8917	0.6903
14	0.0683	− 0.0493	0.3485	− 1.3399
15	− 0.3416	− 0.0493	− 0.5849	1.0012

Calculate the covariance matrix from the data in Table 3, we get:

$${{\varvec{\Sigma}}}^{*} = \left( {\begin{array}{*{20}c} {1} & {{0}{.9814}} & {0.9747} & { - 0.1590} \\ {0.9814} & {1} & {0.9510} & {{ - 0}{.0490}} \\ {0.9747} & {0.9510} & {1} & {{ - 0}{.2730}} \\ { - 0.1590} & {{ - 0}{.0490}} & {{ - 0}{.2730}} & {1} \\ \end{array} } \right)$$

### Establishment of diagnostic model

Calculate the eigenvalues and eigenvectors of the covariance matrix Σ^*^ and sort all the eigenvalues and eigenvectors in descending order of the eigenvalues, as shown in the second and third columns in Table 4. On this basis, calculate the cumulative contribution rate of the variance of the first 1 to 4 eigenvalues, as shown in the fourth column in Table 4.

**Table 4:** Eigenvalues, eigenvectors of the covariance matrix and cumulative contribution of variance.

No	Eigenvalues	Eigenvectors	Cumulative contribution rate of variance
1	2.9772	(− 0.5751, − 0.5653, − 0.5747, 0.1396)^*T*^	74.43%
2	0.9873	(0.0835, 0.1953, − 0.0384, 0.9764)^*T*^	99.11%
3	0.0236	(− 0.2351, − 0.5326, 0.7976, 0.1580)^*T*^	99.70%
4	0.0118	(0.7791, − 0.5989, − 0.1793, 0.0461)^*T*^	100%

As can be seen from Table 4, the first two eigenvalues of the covariance matrix, which have a cumulative contribution rate of variance of 99.11%, are already much higher than the empirical threshold of 80–85%, so let *m* = 2 to construct an approximation of the factor loading matrix ***A*** from the first two eigenvectors.

$$\begin{aligned} & {\mathbf{A}} = \left( {\begin{array}{*{20}c} { - 0.9923} & {0.0829} \\ { - 0.9754} & {0.1941} \\ { - 0.9916} & { - 0.0382} \\ {0.2409} & {0.9702} \\ \end{array} } \right) \\ & \quad \left\{ {\begin{array}{*{20}l} {y_{1}^{*} = - 0.9923F_{1} + 0.0829F_{2} } \hfill \\ {y_{2}^{*} = - 0.9754F_{1} + 0.1941F_{2} } \hfill \\ {y_{3}^{*} = - 0.9916F_{1} - 0.0382F_{2} } \hfill \\ {y_{4}^{*} = 0.2409F_{1} + 0.9702F_{2} } \hfill \\ \end{array} } \right. \\ \end{aligned}$$

It can be seen that there is a large degree of dependence between components $y_{1}^{*}$,$y_{2}^{*}$,$y_{3}^{*}$ and factor *F*_1_, and a smaller degree of dependence with factor *F*_2_, so these 3 quality components are grouped together; $y_{4}^{*}$ is only correlated with factor *F*_2_ to a large extent, and therefore will be divided into a group alone. The final result of the grouping is *G*_1_ = {$y_{1}^{*}$,$y_{2}^{*}$,$y_{3}^{*}$}, *G*_2_ = {$y_{4}^{*}$}.

For group* G*_1_, T^2^ control charts K12, K13 and K23 are built to monitor the binary correlations shift of component pairs ($y_{1}^{*}$,$y_{2}^{*}$), ($y_{1}^{*}$,$y_{3}^{*}$) and ($y_{2}^{*}$,$y_{3}^{*}$); Since there is only one quality component in *G*_2_, there is no need to create a T^2^ control chart; Finally, T^2^ control chart *K* that monitors the correlation shift of all the quality components is established, and the 4 control charts are used to form a diagnostic model of the correlation between the 4 quality components in blade processing.

### Manufacturing process diagnosis

**Figure 8:**
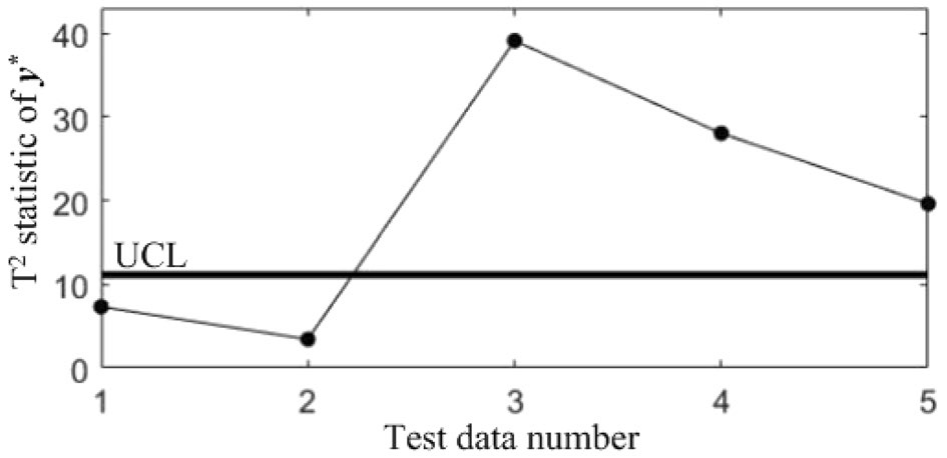
T^2^ control chart *K* for test samples.

In subsequent manufacturing, 5 quality data at different moments are collected, as shown in Table 5, and the results after standardization are shown in Table 6. The T^2^ statistics for the 5 data were calculated and plotted in the T^2^ control chart *K*, as shown in Fig. 8. It can be seen that in the last three samples, the correlations of all the quality components are abnormal.

**Table 5:** Test data collected in subsequent manufacturing.

No	Blade contouring error before matching (*y*_1_/mm)	Blade contouring error after matching (*y*_2_/mm)	Blade positional error (*y*_3_/mm)	Blade torsion error (*y*_4_/’)
1	0.085	0.046	0.098	2.176
2	0.077	0.037	0.092	2.148
3	0.082	0.040	0.103	2.068
4	0.085	0.042	0.095	2.312
5	0.074	0.036	0.086	2.277

**Table 6:** Test data after standardization.

No	y*_1_	y*_2_	y*_3_	y*_4_
1	0.6831	0.8758	0.3485	− 0.2713
2	− 0.9564	− 0.7895	− 0.7716	− 0.5433
3	0.0683	− 0.2344	1.2819	− 1.3204
4	0.6831	0.1357	− 0.2116	1.0497
5	− 1.5712	− 0.9745	− 1.8917	0.7098

**Figure 9:**
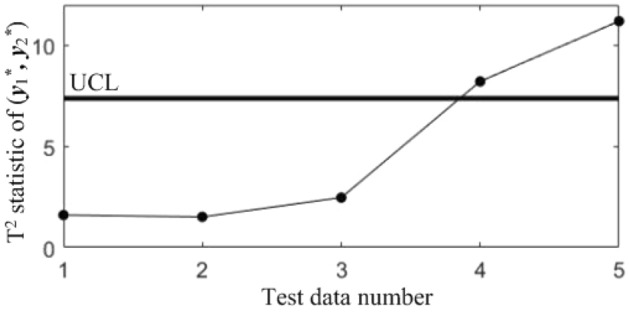
T^2^ control chart K12 monitoring the correlation between *y*_1_^*^ and *y*_2._^*^

**Figure 10:**
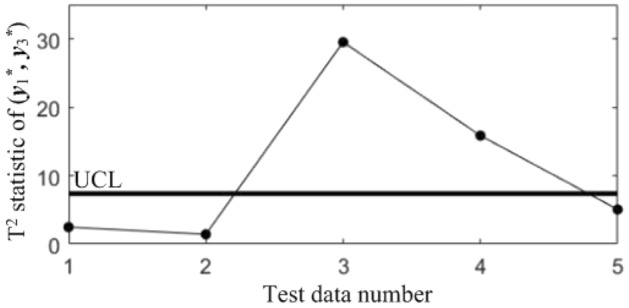
T^2^ control chart K13 monitoring the correlation between *y*_1_^*^ and *y*_3._^*^

**Figure 11:**
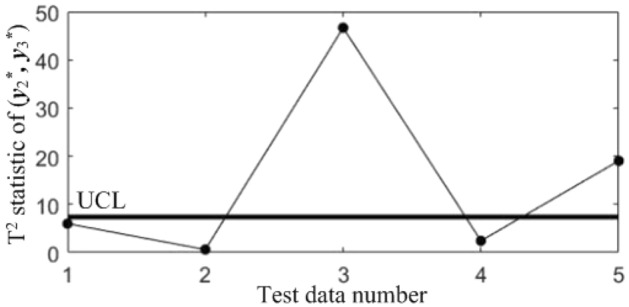
T^2^ control chart K23 monitoring the correlation between *y*_2_^*^ and *y*_3._^*^

In order to diagnose the cause(s) of the abnormal control chart *K*, 3 control charts monitoring the binary correlation shift of the 3 component pairs shown from Figs. 9, 10 and 11 were analyzed, and the diagnostic results are shown in Table 7.

**Table 7:** Diagnostic results.

No	Diagnostic conclusions
1	Normal
2	Normal
3	Anomalous correlations of component pairs (y*_1_, y*_3_), (y*_2_, y*_3_)
4	Anomalous correlations of component pairs (y*_1_, y*_2_), (y*_1_, y*_3_)
5	Anomalous correlations of component pairs (y*_1_, y*_2_), (y*_2_, y*_3_)

### Validity analysis of diagnostic conclusions

In order to judge the accuracy of the diagnostic results in Table 7, another diagnostic model using the CCBD method is built, which contains a total of $C_{4}^{2} + C_{4}^{3} = 10$ T^2^ control charts, as shown in Figs. 12, 13, 14, 15, 16, 17, 18, 19, 20 and 21.

**Figure 12:**
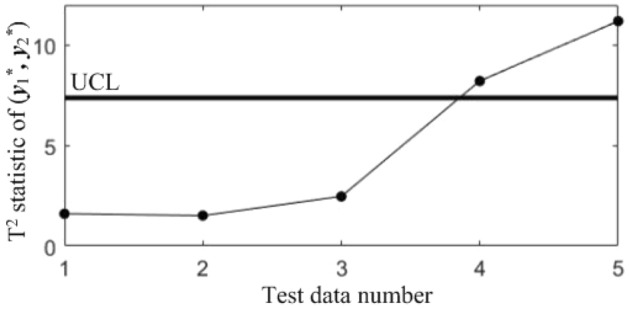
Diagnostic model using CCBD method: T^2^ control chart *KC*_12_ for (y^*^_1_, y^*^_2_).

**Figure 13:**
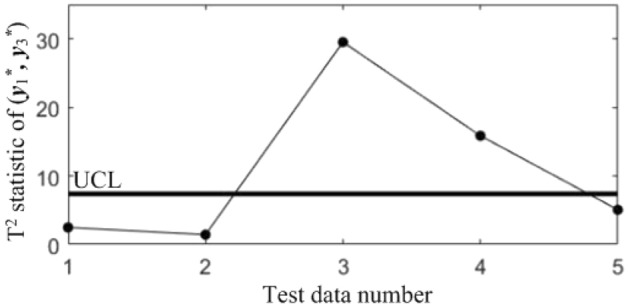
Diagnostic model using CCBD method: T^2^ control chart *KC*_13_ for (y^*^_1_, y^*^_3_).

**Figure 14:**
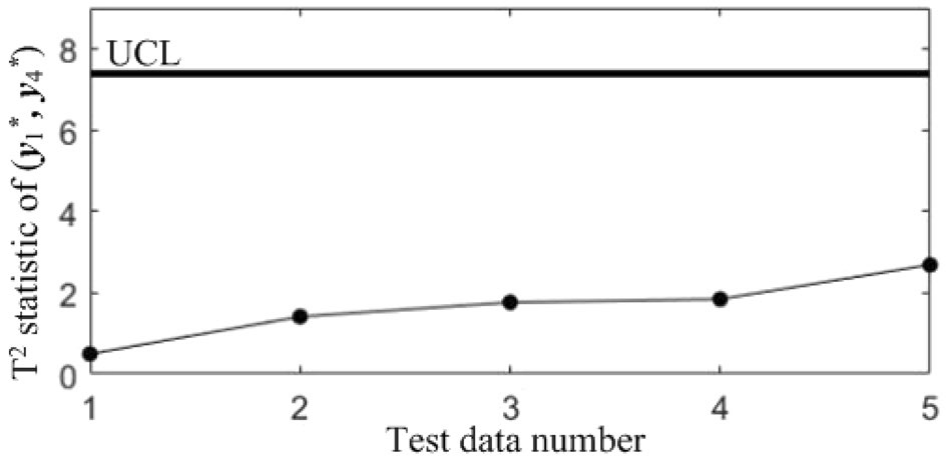
Diagnostic model using CCBD method: T^2^ control chart *KC*_14_ for (y^*^_1_, y^*^_4_).

**Figure 15:**
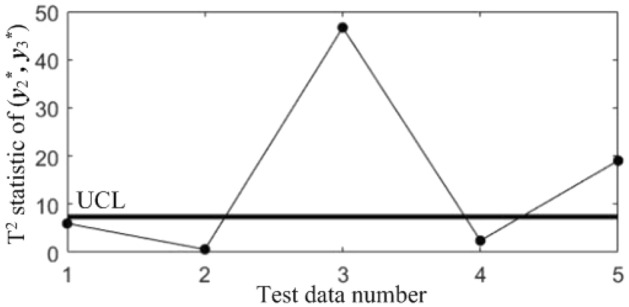
Diagnostic model using CCBD method: T^2^ control chart *KC*_23_ for (y^*^_2_, y^*^_3_).

**Figure 16:**
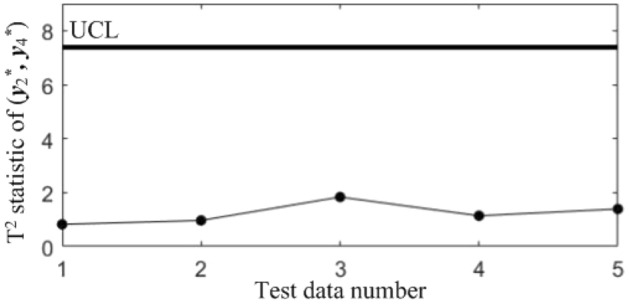
Diagnostic model using CCBD method: T^2^ control chart *KC*_24_ for (y^*^_2_, y^*^_4_).

**Figure 17:**
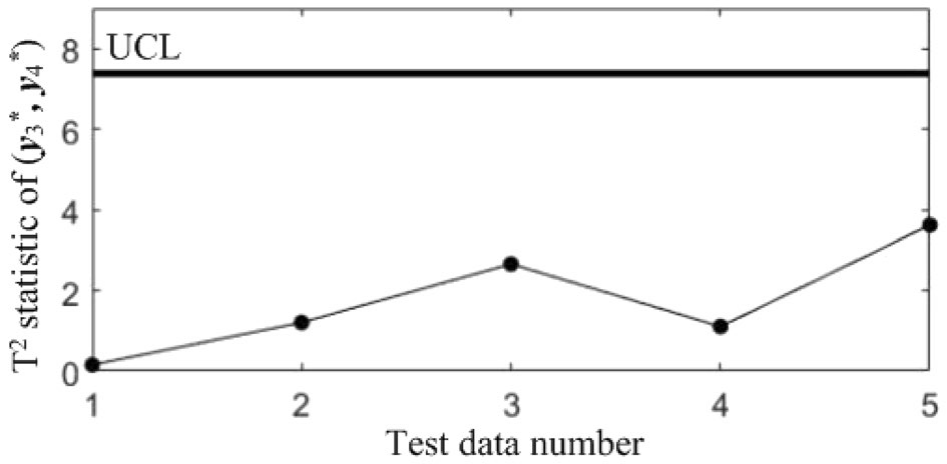
Diagnostic model using CCBD method: T^2^ control chart *KC*_34_ for (y^*^_3_, y^*^_4_).

**Figure 18:**
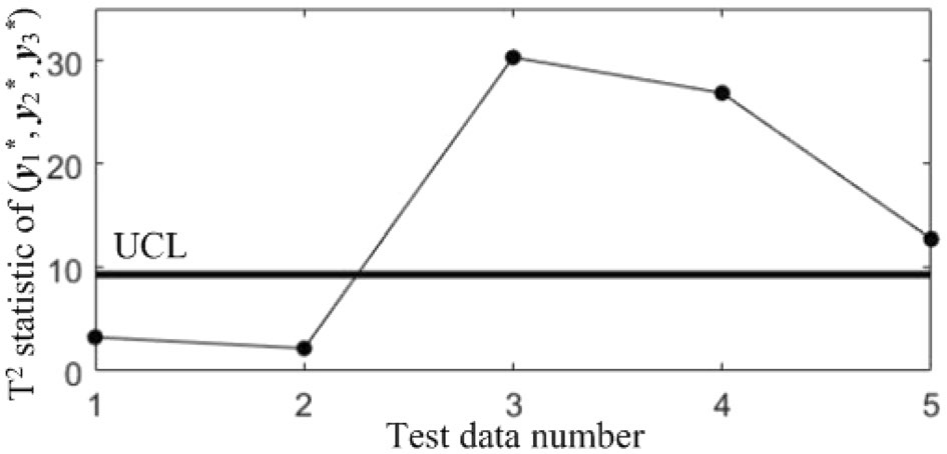
Diagnostic model using CCBD method: T^2^ control chart *KC*_123_ for (y^*^_1_, y^*^_2_, y^*^_3_).

**Figure 19:**
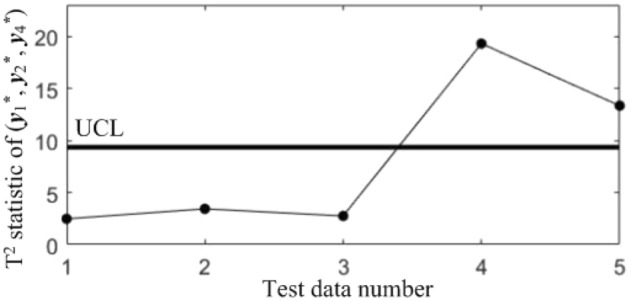
Diagnostic model using CCBD method: T^2^ control chart *KC*_124_ for (y^*^_1_, y^*^_2_, y^*^_4_).

**Figure 20:**
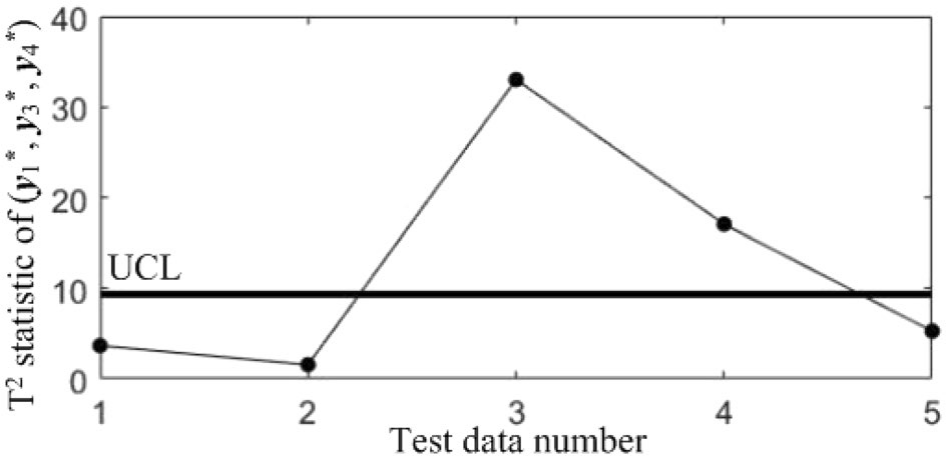
Diagnostic model using CCBD method: T^2^ control chart *KC*_134_ for (y^*^_1_, y^*^_3_, y^*^_4_).

**Figure 21:**
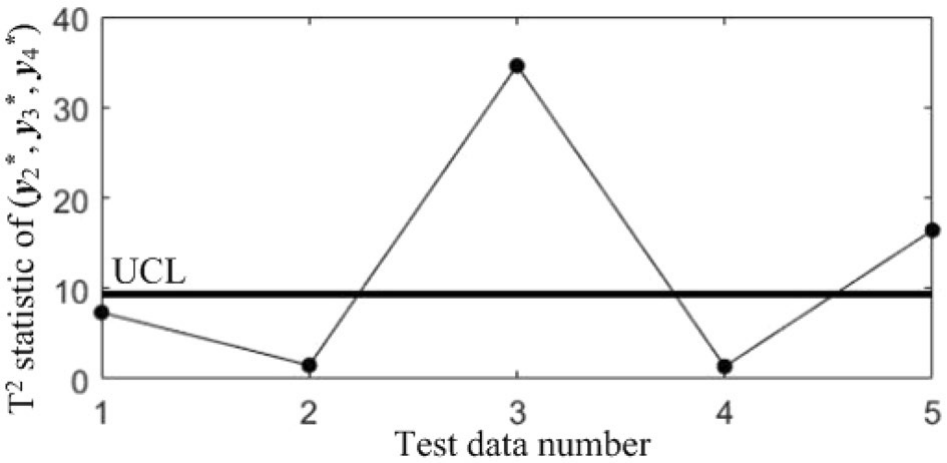
Diagnostic model using CCBD method: T^2^ control chart *KC*_234_ for (y^*^_2_, y^*^_3_, y^*^_4_).

In order to compare the diagnostic conclusions derived from the two different diagnostic models, they are placed in Table 8. It can be seen that there are differences in the diagnostic conclusions of the last 3 points. Taking point 3 as an example, further analysis of the diagnostic results using the CCBD method reveals that since the correlations of component pairs (y^*^_1_, y^*^_3_), (y^*^_2_, y^*^_3_) are anomalous, the correlations of other component combinations containing (y^*^_1_, y^*^_3_) or (y^*^_2_, y^*^_3_) are bound to be in anomalous states, and thus the diagnostic results that the correlation abnormalities of component combinations (y^*^_1_, y^*^_2_, y^*^_3)_, (y^*^_1_, y^*^_3_, y^*^_4_) and (y^*^_2_, y^*^_3_, y^*^_4_) are redundant diagnostic information. After removing the redundant diagnostic information, the diagnostic results of both diagnostic models for the causes of the anomaly in point 3 are identical. A similar analysis of the diagnostic results for points 4 and 5 leads to the same conclusions, as shown in Table 9. Therefore, the accuracy of the diagnostic method of multivariate process quality correlation based on the grouping technique can be guaranteed.

**Table 8:** Comparison of the diagnostic results of the two diagnostic models.

No	Grouping-based diagnostic model	Diagnostic model using CCBD method
1	Normal	Normal
2	Normal	Normal
3	Anomalous correlations of component pairs (y*_1_, y*_3_), (y*_2_, y*_3_)	Anomalous correlations of component combinations (y*_1_, y*_3_), (y*_2_, y*_3_), (y*_1_, y*_2_, y*_3_), (y*_1_, y*_3_, y*_4_), (y*_2_, y*_3_, y*_4_)
4	Anomalous correlations of component pairs (y*_1_, y*_2_), (y*_1_, y*_3_)	Anomalous correlations of component combinations (y*_1_, y*_2_), (y*_1_, y*_3_), (y*_1_, y*_2_, y*_3_), (y*_1_, y*_2_, y*_4_), (y*_1_, y*_3_, y*_4_)
5	Anomalous correlations of component pairs (y*_1_, y*_2_), (y*_2_, y*_3_)	Anomalous correlations of component combinations (y*_1_, y*_2_), (y*_2_, y*_3_), (y*_1_, y*_2_, y*_3_), (y*_1_, y*_2_, y*_4_), (y*_2_, y*_3_, y*_4_)

**Table 9:** Comparison of the two diagnostic systems after redundant diagnostic results are removed.

No	Grouping-based diagnostic model	Diagnostic model using CCBD method
1	Normal	Normal
2	Normal	Normal
3	Anomalous correlations of component pairs (y*_1_, y*_3_), (y*_2_, y*_3_)	Anomalous correlations of component pairs (y*_1_, y*_3_), (y*_2_, y*_3_)
4	Anomalous correlations of component pairs (y*_1_, y*_2_), (y*_1_, y*_3_)	Anomalous correlations of component pairs (y*_1_, y*_2_), (y*_1_, y*_3_)
5	Anomalous correlations of component pairs (y*_1_, y*_2_), (y*_2_, y*_3_)	Anomalous correlations of component pairs (y*_1_, y*_2_), (y*_2_, y*_3_)

## Discussion and conclusion

For the problem of correlation diagnosis in multivariate process quality management, this paper proposed a grouping technique based correlation diagnosis method. Compared with the present diagnostic methods, the method proposed in this paper has the following advantages:

1.1. The diagnosis is more efficient

The space complexity of the multivariate process quality correlation diagnostic method based on grouping technique is approximately *O*(*p*), while the space complexity of the diagnostic algorithm based on the CCBD method, principal component analysis method and the orthogonal decomposition of the T^2^ statistic are *O*(2^*p*^), *O*(*p*^2^), and *O*(*p*!), respectively. Therefore, the proposed method in this paper has higher diagnostic efficiency.

2.2. The diagnostic results are more accurate

The grouping technique based multivariate process quality correlation diagnosis method takes the correlation of component pairs as the diagnostic unit. Because component pairs are the minimum combination of quality components, the disadvantage of redundant diagnostic information in diagnostic algorithms based on the CCBD method, the principal component analysis method and the orthogonal decomposition of T^2^ statistics can be avoided to provide more accurate diagnostic results for manufacturing processes.

3.3. Better generality

Compared with the diagnostic methods based on artificial intelligence technology, the diagnostic method proposed in this paper is based on strict mathematical analysis as the theoretical foundation, avoids the defect of intelligent diagnostic methods in which the network structure and parameters are oriented to specific application. Therefore, the proposed method can be used as a general theoretical model for the multivariate process quality correlation diagnosis.

The multivariate process quality diagnostic model based on grouping technique has the following two issues for further discussion in its application.

- (1) Judgment of the difference degree in correlations between quality components

The difference degree in correlations between quality components can be judged by the covariance matrix Σ^*^ obtained after standardizing the quality data collected in stable state. In general, if there exists at least one row of elements in Σ^*^ such that the ratio of the maximum value to the minimum value, except for the main diagonal element, is not less than 2, it can be tentatively determined that there is a large difference in the correlations between different quality components.

- (2) Basis for grouping quality components

The maximum value of each row elements in the factor loading matrix ***A*** can be used as a basis for grouping the quality components. The quality component *y*_i_^*^ can be assigned to group *G*_*k*_ represented by the common factor *F*_*k*_ if *a*_*ik*_ is the element with the largest absolute value in the *i*th row of ***A***. Experience has shown that grouping is more desirable when *a*_*ik*_ > 0.7. When the difference between the absolute values of the elements of a row in ***A*** is small, it indicates that the corresponding quality component has an approximately equal degree of dependence on all the common factors, and at this point, the group where the corresponding quality component is located can be rationally determined in conjunction with the actual interpretation of the factor analysis model. If the absolute values between the elements of any row in ***A*** are all approximately equal, it indicates that the degree of dependence of all quality components on all common factors is approximately equal, at this time, all quality components are located within a same group, and the diagnostic model is degraded to the diagnostic method based on the correlation decomposition. We will study this issue in depth in our later work.

## Ethics declarations

The authors declare no human or animal subjects, sample or database was used in this manuscript.

### Supplementary Information

Supplementary Information.

## Data Availability

All data generated or analyzed during this study are included in this manuscript.
