# Supplementary material for: A multivariate process quality correlation diagnosis method based on grouping technique
Source: Sci Rep. 2024 Jun 8;14:13212. doi: 10.1038/s41598-024-61954-y (PMC11162481; doi:10.1038/s41598-024-61954-y)
Supplement: Supplementary file 1 — Supplementary Information. [file 41598_2024_61954_MOESM1_ESM.doc]

**Supplementary**

Suppose the multivariate process quality ***y***=(*y*_1_, *y*_2_,…, *y_p_*)*^T^* follows the normal distribution *N*(***μ***, ***Σ***), then its T^2^ statistic follows the *χ*^2^ distribution with *p* freedom.

*Proof*. The T^2^ statistic is defined as:

$$T^{2}=(\mathbf{y}-\mathbf{\mu})^{T}\mathbf{\Sigma}^{-1}(\mathbf{y}-\mathbf{\mu})$$

It follows from Theorem 3 in the main text that ***Σ***^-1^ is a real symmetric positive definite matrix. A square root decomposition of it gives:

$$\mathbf{\Sigma}^{-1}=\left(\mathbf{\Sigma}^{-\frac{1}{2}}\right)^{T}\mathbf{\Sigma}^{-\frac{1}{2}}$$

Let $\mathbf{z}=\mathbf{\Sigma}^{-\frac{1}{2}}(\mathbf{y}-\mathbf{\mu}{})$, we get:

$$\frac{\partial\mathbf{y}}{\partial\mathbf{z}}=\mathbf{\Sigma}^{\frac{1}{2}}$$

According to the method of calculating the probability density function of a random vector function, the probability density function of the random vector ***z*** is:

The above shows that the joint probability density function of the random vector ***z*** can be expressed as the product of the probability density functions of the components, so the components in ***z*** are independent of each other and all follow the standard normal distribution *N*(0, 1). At this point, the T^2^ statistic can be expressed as:

$$T^{2}=(\mathbf{y}-\mathbf{\mu})^{T}\left(\mathbf{\Sigma}^{-\frac{1}{2}}\right)^{T}\mathbf{\Sigma}^{-\frac{1}{2}}(\mathbf{y}-\mathbf{\mu})=\mathbf{z}^{T}\mathbf{z}=\sum_{i=1}^{p} z_{i}^{2}$$

By the definition of the *χ*^2^ statistic, the T^2^ statistic at this point follows the *χ*^2^ distribution with *p* freedom.
